# Vector-borne pathogen profiling and phylogeny of ticks infesting domestic dogs in Chad, Africa, using targeted next-generation sequencing and DNA barcoding

**DOI:** 10.1186/s13071-026-07566-z

**Published:** 2026-07-16

**Authors:** Ian Kirimi Daniel, Maureen A. Kelly, Rafael A. N. Ramos, Hassan Hakimi, Jobin Jose Kattoor, Rebecca P. Wilkes, Metinou K. Sidouin, Richard Ngandolo Bongo Nare, Philip Tchindebet Oaukou, Meriam N. Saleh, Ellen Haynes, Christopher A. Cleveland, Michael J. Yabsley, Rebecca Garabed, Lucienne Tritten, Guilherme G. Verocai

**Affiliations:** 1 Department of Veterinary Pathobiology, College of Veterinary Medicine and Biomedical Sciences, Texas A&M University, College Station, TX USA; 2 Laboratory of Parasitology, Federal University of the Agreste of Pernambuco, Garanhuns, PE Brazil; 3 Department of Diagnostic Medicine and Pathobiology, College of Veterinary Medicine, Kansas State University, Manhattan, KS USA; 4 Department of Infectious Diseases and Microbiology, School of Public Health, University of Pittsburgh, Pittsburgh, PA USA; 5 Department of Comparative Pathology, College of Veterinary Medicine, Purdue University, West Lafayette, IN USA; 6 Department of Veterinary Science and Veterinary Diagnostic Laboratory, University of Kentucky, Lexington, KY USA; 7 The Carter Center–Chad, N’Djamena, Chad; 8 Institut de Recherche en Elevage pour le Développement (IRED), Afrique One REACH, N’Djamena, Chad; 9 Chad Ministry of Public Health; Programme National d’Eradication du Ver de Guinée, (National Guinea Worm Eradication Program–Chad), N’Djamena, Chad; 10 Southeastern Cooperative Wildlife Disease Study, Department of Population Health, College of Veterinary Medicine, University of Georgia, Athens, GA USA; 11 Center for Ecology of Infectious Diseases, University of Georgia, Athens, GA USA; 12 Warnell School of Forestry and Natural Resources, University of Georgia, Athens, GA USA; 13 Department of Veterinary Preventive Medicine, College of Veterinary Medicine, The Ohio State University, Columbus, OH USA; 14 Institute of Parasitology, Faculty of Agricultural and Environmental Sciences, McGill University, Sainte-Anne-de-Bellevue, QC Canada

**Keywords:** Africa, Ixodidae, One Health, Targeted next-generation sequencing, Tick-borne pathogens

## Abstract

**Background:**

Understanding the range of vectors and pathogen diversity in sub-Saharan Africa is crucial for efficient disease prevention and control efforts. Domestic dogs are susceptible to many vector-borne pathogens (VBPs) and serve as sentinels for several human pathogens. Yet, critical surveillance gaps in canine VBPs persist in Central Africa, where the tropical climate and pastoral agro-livelihoods create unique transmission risks.

**Methods:**

We conducted a cross-sectional survey from September to October 2021, collecting 4238 ticks from 1254 domestic dogs across 56 villages in Chad, Africa. All ticks were identified morphologically and/or by sequence analysis of 16S rDNA*,* 12S rDNA*,* and cytochrome c oxidase subunit I *(COI)* gene regions. A representative subset (*n* = 618) was subsequently screened for comprehensive, simultaneous detection of 21 VBPs using a targeted next-generation sequencing (tNGS) assay. Logistic regression models were used to assess associations between potential risk factors and pathogen detection.

**Results:**

Five tick species belonging to four genera were identified: *Rhipicephalus linnaei* (89.8%; 3805/4238)*, Rhipicephalus muhsamae* (9.4%; 400/4238)*, Amblyomma variegatum* (0.6%; 26/4238), *Haemaphysalis leachi* (0.1%; 4/4238), and *Hyalomma truncatum* (0.1%; 3/4238). Overall, 59.1% (365/618; 95% CI 55.1 – 62.9%) of the ticks were positive for at least one pathogen. We detected 14 pathogens, with the most prevalent being *Hepatozoon canis* (47.6%,* n* = 294/618), followed by *Ehrlichia canis* (8.4%, *n* = 52/618), *Coxiella*-like endosymbionts (5.3%, *n* = 33/618), *Rickettsia* spp. (4.7%, *n* = 29/618), *Anaplasma platys* (4.4%, *n* = 27/618), *Anaplasma* spp. (3.7%, *n* = 23/618), hemotropic *Mycoplasma* spp. (1.5%, *n* = 9/618), and *Anaplasma phagocytophilum* (1.1%, *n* = 7/618). *Babesia caballi, Hepatozoon felis, Rickettsia conorii,* and *Rickettsia massiliae* were each identified in < 1% of the ticks. The highest pathogen diversity was detected in *R. linnaei*, although *R. muhsamae* harbored the highest diversity of zoonotic *Rickettsia* species. Independent predictors for VBP infections included tick species for *E. canis* (*P* = 0.02) and *H. canis* (*P* = 0.001), and geographic region for* E. canis* (*P* = 0.01).

**Conclusions/Significance:**

This study provides a comprehensive molecular characterization of canine VBPs in ticks from Central Africa using deep sequencing. The high pathogen prevalence and diversity highlight the need for expanded surveillance and control strategies in Chad, including educating dog owners on ectoparasite prevention.

**Graphical Abstract:**

**Figure:**
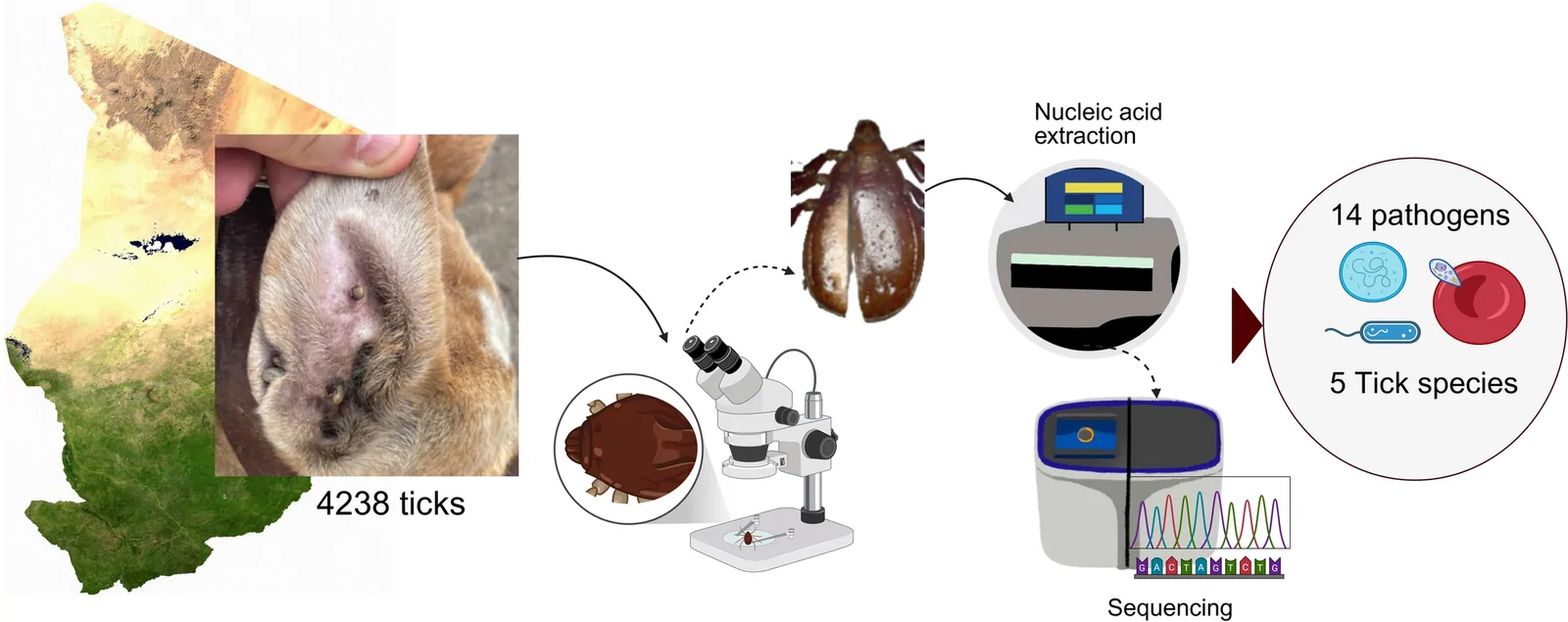

**Supplementary Information:**

The online version contains supplementary material available at https://doi.org/10.1186/s13071-026-07566-z.

## Background

Vector-borne pathogens (VBPs) are recognized as ubiquitous across the African continent. They are transmitted by a diverse range of arthropod vectors including ticks (*Anaplasma* spp., *Ehrlichia* spp., *Hepatozoon* spp., *Rickettsia* spp., *Babesia* spp.), mosquitoes (*Dirofilaria* spp.), and fleas (hemotropic mycoplasmas), posing significant threats to animal and human health [1, 2]. Infections with VBPs may result in significant clinical manifestations from direct effects of the pathogen itself and host immunopathological responses, leading to potentially fatal outcomes.

Tick-borne pathogens (TBPs) are considered the second most significant vector-borne threat to human health worldwide [3]. Because dogs live in close association with humans and often interact with wildlife and livestock in sub-Saharan Africa, they occupy a unique position in the epidemiological cycle of these pathogens [4]. This close connection makes dogs both vulnerable to TBP-related morbidity and mortality and valuable as sentinel species for monitoring zoonotic disease circulation. Consequently, integrated One Health surveillance approaches that include dogs are essential for understanding and managing the broader ecological dynamics of vector-borne pathogens [5, 6].

Ticks can transmit a wide range of pathogenic agents, including bacteria, viruses, and parasites [7]. In sub-Saharan Africa, molecular surveillance in both ticks and their canine hosts has revealed substantial geographic variation in VBP prevalence [8]. In a study in Chad, *H. canis* prevalence in dogs ranged from 67.2 to 93.4%, *E. canis* from 7.0 to 27.8%, and *A. platys* from 10.1 to 22.0%, with *Rickettsia* spp. detected in 43.3% of ticks from these dogs by PCR, demonstrating variable pathogen-host dynamics across the region [9]. However, systematic surveillance using advanced high-throughput molecular methods remains absent from Chad, creating gaps in understanding regional pathogen circulation and zoonotic risk assessment. Furthermore, knowledge of resident tick species remains scarce, with only a single report focusing on ticks infesting domestic dogs in Chad [10].

The single previous survey in Chad documented dog infestations by multiple tick genera, including *Amblyomma, Haemaphysalis, Hyalomma,* and *Rhipicephalus* [10]. The genus *Rhipicephalus*, comprising over 70 species, represents the predominant hard tick genus in sub-Saharan Africa [11]. Although the genus is common in Africa, the brown dog tick *Rhipicephalus sanguineus* s.l. is present on dogs globally [11, 12] and is responsible for the transmission of numerous pathogens, including *A. platys*, *Babesia vogeli*, *E. canis*, *H. canis*, and *Rickettsia* spp., [13–15], and is a suspected vector of hemotropic *Mycoplasma* species [16]. The yellow tick, *Haemaphysalis leachi*, is the other species primarily adapted to feeding on domestic dogs in Africa [17]. In contrast, *Amblyomma variegatum* and *Hyalomma truncatum* are primarily ticks of large ungulates [17], although they infrequently parasitize dogs and can contribute to the ecology of VBPs in regions where dogs and livestock coexist. Furthermore, ticks can harbor multiple pathogens simultaneously, leading to coinfections or codetections with the potential for concurrent transmission to hosts [18].

Effective surveillance and control of VBPs and related diseases in humans and domestic animals requires accurate identification and mapping of tick vectors, along with precise diagnosis of their pathogens. Surveillance for VBPs in Chad has predominantly employed conventional diagnostic approaches, including PCR and immunological assays [9, 19–21]. However, these traditional methodologies present significant limitations that constrain comprehensive pathogen assessment. Traditional DNA-based methods, although valuable, are inherently restricted by their targeted approach, typically accommodating only three to four specific targets per assay [22]. Immunological assays, including enzyme-linked immunosorbent assay (ELISA), indirect immunofluorescence assay (IFA), commercial point-of-care (POC) tests, and immunoblot techniques, detect host antibody responses to VBP exposure, but are incapable of differentiating past and ongoing infections [23]. This limitation is particularly problematic in endemic regions where repeated pathogen exposure may result in persistent antibody levels that confound diagnostic interpretation [23, 24]. Additionally, the specificity of immunological methods can be compromised by cross-reactivity between closely related pathogens, leading to potential false-positive results [24].

The emergence of next-generation sequencing (NGS) and targeted metagenomic approaches provide new opportunities for pathogen discovery and genomic surveillance [25]. Specifically, the new technology of targeted next-generation sequencing (tNGS) for canine VBPs [26] is a sensitive and specific technique that allows for simultaneous detection of up to 21 canine VBPs in a single run while providing sequence confirmation. The assay enables comprehensive VBP surveillance, including the identification of novel or unexpected pathogens that may be missed by targeted PCR assays [26]. Initial application of tNGS by our group in this region revealed high prevalence rates and coinfections involving multiple pathogens in the blood of domestic dogs [27]. However, comparable studies assessing VBPs within tick vectors using deep sequencing remain absent. Hence, this study aimed to (1) expand knowledge of the diversity of ticks infesting rural domestic dogs in Chad, Africa, (2) apply a tNGS assay to explore and characterize the diversity of VBPs in ticks infesting domestic dogs, and (3) analyze coinfection patterns and predictors of those VBPs using logistic regression models.

## Methods

### Study location and tick collection

**Fig. 1:**
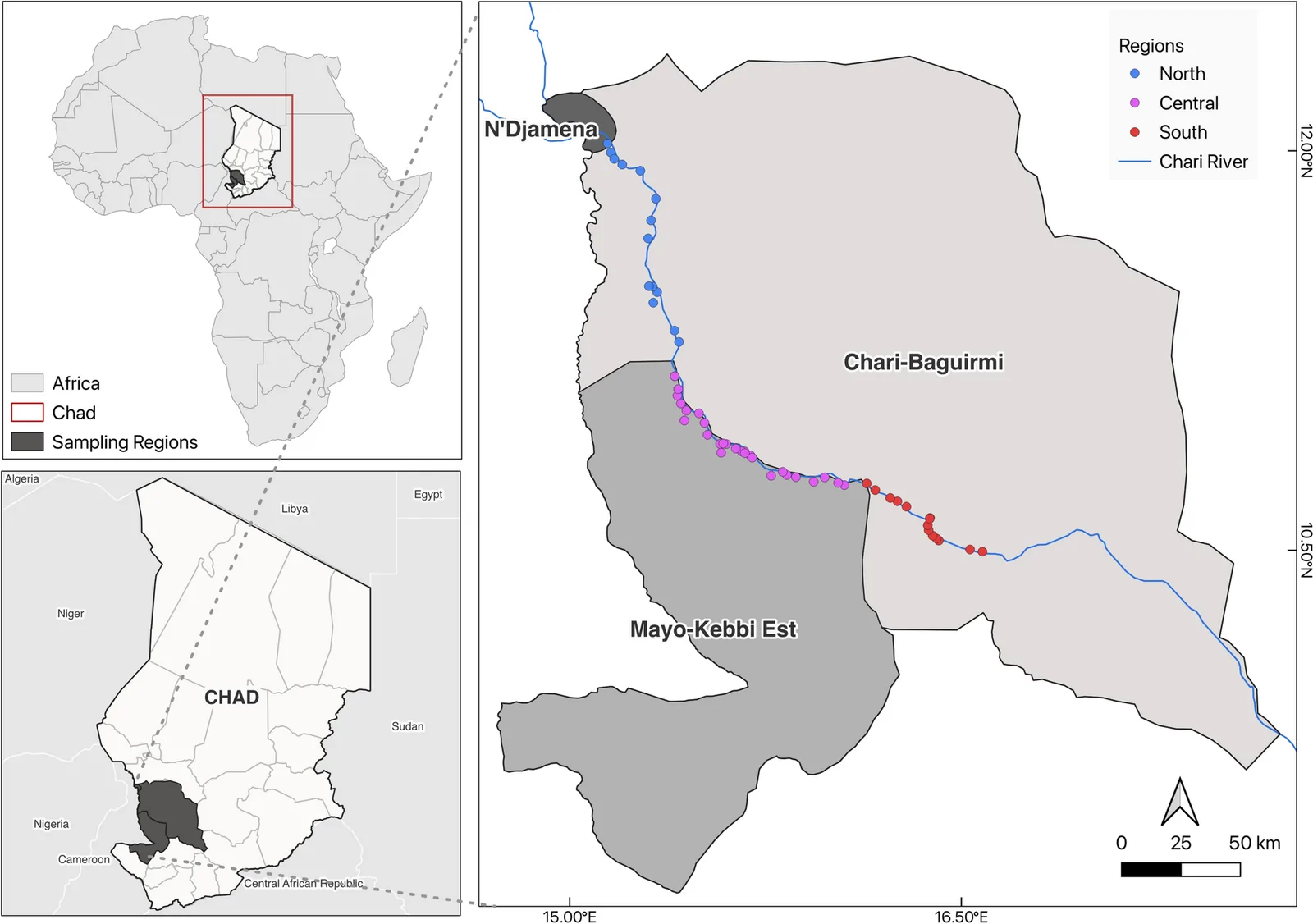
Map showing the 56 villages along the Chari River where ticks were collected from domestic dogs in 2021. The colored dots represent the village clusters based on climate variability; Blue = North, Purple = Central and Red = South. The map was generated using the open-source Geographic Information System software (QGIS) version 3.2.2

Ticks (*n* = 4238) were collected from 1254 domestic dogs residing in 56 villages along the Chari River in two administrative regions of Chad; Chari Baguirmi and Mayo Kebbi Est. The study site was stratified into northern, central, and southern zones based on the distinct climatic gradients: the southern region features a tropical savanna climate, the central zone presents a transitional semi-arid climate with moderate temperatures and seasonal rainfall, while the northern zone exhibits drier conditions with extreme aridity, elevated temperatures, and low humidity [28]. Specimens were collected from September to October 2021, along the Chari River, coinciding with the end of the rainy season. This collection was conducted as part of a concurrent study of Guinea worm (*Dracunculus medinensis*) with the support of the Programme National d’Eradication du Ver de Guinée – Tchad. A detailed map illustrating the study region and sampled villages (Fig. 1) has been adapted from Daniel* et al.* 2025 [27].

Upon collection, ticks were preserved in 70% ethanol and subjected to a water bath treatment at 60 °C for approximately 15 min to eliminate potential foot and mouth disease virus (FMDV) before shipping to the United States (USA) under USDA permit 610–22–278–03,572. Metadata were recorded for each specimen, including host demographics (i.e., sex and approximate age) and the geographic region.

### Tick processing and morphological identification

A total of 4238 ticks were collected, most of which were identified to species level using appropriate morphological keys [17, 29, 30]. However, molecular confirmation via DNA barcoding was performed for 26 specimens where morphological features were compromised or insufficient, particularly for species belonging to complexes, including *Rhipicephalus muhsamae* (part of the *Rhipicephalus simus* complex) and *R. sanguineus* s.l. (belonging to a group of at least 16 morphologically similar brown dog tick species).

### Tick selection for pathogen screening

For comprehensive VBP screening, a stratified sampling approach was implemented to ensure representative pathogen surveillance across the study population. The overwhelming predominance of *R. linnaei* (formerly the tropical lineage of *R. sanguineus* s.l.) in our collection, coupled with limited female representation among less abundant species (*R. muhsamae*, *A. variegatum*, *Ha. leachi, and Hy. truncatum*), necessitated this approach to maintain adequate sample sizes for comparative analyses across tick species. The sampling protocol focused exclusively on female tick specimens based on methodological considerations. Female ticks exhibit prolonged host attachment and consume substantially greater blood volumes than males, characteristics that enhance both the probability of pathogen transmission and the analytical sensitivity of pathogen detection [31, 32]. Second, restricting analysis to a single sex eliminates potential sex-related variation in pathogen prevalence reported in some studies [33, 34], thereby reducing confounding variables in our statistical analyses. To mitigate host-related clustering effects although maintaining statistical power, sampling was further limited to one tick per dog using the following criteria: (1) For *R. linnaei*, 30% of the dogs were randomly selected using computer-generated random numbers from each of the villages with > 10 infested dogs. In villages with ≤ 10 infested dogs, all dogs were included. When multiple female ticks were present on selected dogs, one specimen was randomly selected, and (2), for the remaining species (*R. muhsamae, A. variegatum, Ha. leachi,* and *Hy. truncatum*), all dogs infested with these species were represented, with random selection of a single female tick, per species, applied when multiple specimens occurred on the same host.

This systematic approach yielded 618 female ticks with balanced geographic and host distribution although controlling for potential confounding associated with pseudo-replication from the same host. These 618 ticks were subsequently subjected to nucleic acid extraction and pathogen screening as described below. Of the total 4,238 collected specimens, 26 ticks were additionally subjected to DNA barcoding for molecular confirmation of species where morphological features were compromised or insufficient, as described in the tick processing and morphological identification section.

### Nucleic acid extraction

A total of 618 ticks were individually surface cleaned with a 10% household bleach solution (≈5.25% sodium hypochlorite, NaOCl; Clorox Company, Oakland, CA, USA), followed by brief rinses in 70% ethanol and distilled water, then dried on sterile paper towels. Using sterile scalpel blades, whole ticks were dissected into quarters, of which all were then digested in 300 µL of Tissue Lysis buffer (TLA) and 30 µL of proteinase K (Promega Corporation, Madison, WI, USA) at 56 °C for 1 h with shaking. For complete disruption, a disposable pestle was used to mechanically macerate ticks after 30 min of shaking, followed by a final 30 min of incubation. The lysate was used for nucleic acid extraction using a Maxwell^®^ RSC Tissue DNA Kit (Promega Corporation, Madison, WI, USA) in the Maxwell^®^ RSC 48 instrument (Promega Corporation, Madison, WI, USA) according to the manufacturer’s specifications. Extracted nucleic acids were stored at –20 °C until analysis.

### Detection of VBPs by tNGS and bioinformatic analysis

Ticks were subjected to comprehensive, simultaneous detection of multiple canine VBPs using a tNGS assay designed to detect 21 pathogen targets, including species of *Anaplasma*, *Ehrlichia*, *Babesia*, *Bartonella*, *Rickettsia*, *Borrelia*, *Leishmania*, *Cytauxzoon* and hemotropic *Mycoplasmas* [26]. VBP detection was performed using validated protocols as previously described [26, 27]**.** Raw pathogen sequencing data were assembled using SPAdes (v5.12.0.0) on the Torrent Server (TSS) and subsequently aligned to the target sequence genomic regions within the TSS. Read evaluation and validation were performed using Geneious Prime Version. 2021.1.1 (https://www.geneious.com/prime/) with additional BLAST searches against the NCBI database (https://blast.ncbi.nlm.nih.gov/Blast.cgi) (Bethesda, MD, USA) to confirm pathogen identification.

### Conventional PCR and species barcoding (ticks)

Conventional PCR (cPCR) was used for species identification using a multi-gene approach targeting the 16S rDNA region as the primary marker, with 12S rDNA serving as a secondary target when primary amplification failed or additional confirmation was required. The *COI* gene was additionally targeted for specimens where both 16S rDNA and 12S rDNA amplification were unsuccessful or inconclusive. All reactions were performed in a 25 µL volume containing 8.75 µL of molecular-grade water, 0.625 µL (10 µM) per primer, 12.5 µL of 2 × GoTaq^®^ Green Master Mix (Promega Corporation, Madison, WI, USA), and 2.5 µL of DNA template. Each PCR run included DNA of *Ornithodoros turicata* (positive control) and molecular-grade water (negative control). PCR amplifications were performed using previously described primer sets and thermal cycling conditions [10, 35–37].

All cPCR products were resolved on 1.5% agarose gels containing GelRed™ Nucleic Acid Gel Stain (GoldBio, St. Louis, MO, USA), and positive bands were visualized using an ultraviolet transilluminator (ChemiDoc Imaging System, BioRad, Hercules, CA, USA). All positive amplicons displaying clear bands of the expected product size were purified using the Cycle Pure E.Z.N.A.^®^ Purification Kit (Omega Bio-Tek Inc., Norcross, GA, USA) following the manufacturer’s recommendations and bidirectionally sequenced using the Eurofins Sanger sequencing service (Eurofins Genomics, Louisville, KY, USA) using the original cPCR primers.

Subsequently, bidirectional sequences were obtained and manually assembled to generate consensus contigs using MEGA X [38]. Contigs were subjected to homology searches against the GenBank database by the Basic Local Alignment Search Tool (BLAST) program (https://blast.ncbi.nlm.nih.gov), retrieving reference sequences for each of the three mitochondrial gene markers. Multiple sequence alignments for each marker were performed independently using MAFFT v7 [39]. Maximum-likelihood (ML) phylogenetic trees were constructed separately for the 12S and 16S gene markers using IQ-TREE v2 [40]. The software’s integrated ModelFinder feature determined and applied the optimal nucleotide substitution model for each individual dataset. Branch support values were evaluated through 1000 ultrafast bootstrap approximation replicates to assess the robustness of the inferred phylogenetic relationships within each tree. The resulting phylogenetic trees were visualized and annotated using Interactive Tree of Life (iTOL) v6 [41]. *Ornithodoros moubata* was designated as the outgroup taxon to root the phylogenetic trees. All newly generated contigs were submitted to GenBank using the BankIt submission tool of the National Center for Biotechnology Information (NCBI) (https://www.ncbi.nlm.nih.gov/genbank/) (Bethesda, MD, USA).

### Statistical analysis

Pathogen data were first managed in Microsoft Excel (version 1808) and then analyzed using R version 4.4.1 and RStudio 2024.09.1 Build 764. Binary outcome variables for VBP presence/absence were created based on the tNGS assay results. Overall positivity rates were calculated as the number of VBP species-infected ticks (individual specimens), divided by the total number of molecularly screened tick specimens (*n* = 618) (Table 1). Descriptive statistics included pathogen-specific infection rates, single and coinfection patterns (Table 2), and estimated true prevalence along with 95% confidence intervals determined by the Wilson method. —﻿C Logistic regression GLMs were fitted using the *lme4* package in R to quantify relationships between explanatory variables (tick species, geographic region) and pathogens with > 10 positive detections: *Anaplasma* spp., *A. platys*, *E. canis*, *H. canis*, and *Rickettsia* spp. Analyses were restricted to *R. linnaei* and *R. muhsamae*, which collectively comprised 97.8% of the collection. *Amblyomma variegatum*, *Ha. leachi*, and *Hy. truncatum* were excluded due to low sample sizes and pathogen prevalence (Table 1), precluding minimum expected cell frequencies for valid chi-square testing [42] and sufficient events-per-variable ratios for stable regression estimates [43]. All predictor variables were included a priori in multivariable logistic regression models without univariable pre-screening to avoid selection bias and preserve statistical power, as univariable filtering may exclude variables that demonstrate significance only in adjusted analyses [44–46]. Univariable logistic regression was still performed separately to examine crude/unadjusted associations between individual predictors and pathogen outcomes (Table 3). Models yielded adjusted odds ratios (aORs) with 95% confidence intervals. Bonferroni correction was applied using the *p. adjust* function in R, to correct for familywise error rate at *α* = 0.05.

**Table 1:** Overall positivity rate for each vector-borne pathogen detected in ticks (*n* =618) infesting dogs from Chad

Pathogen	No. Positive	Positivity rate	95% CI
*Anaplasma platys*	27	4.4	3.0 – 6.3
*Anaplasma phagocytophilum*	7	1.1	0.5 – 2.3
*Anaplasma* spp.	23	3.7	2.5 – 5.5
*Babesia caballi*	1	0.2	0.0 – 0.9
*Cand.* M. haematoparvum	2	0.3	0.1 – 1.2
*Cand.* M. haemominutum	2	0.3	0.1 – 1.2
*Coxiella*-like endosymbionts	33	5.3	3.8 – 7.4
*Ehrlichia canis*	52	8.4	6.5 – 10.9
*Hepatozoon canis*	294	47.6	43.7 – 51.5
*Hepatozoon felis*	1	0.2	0.0 – 0.9
*Mycoplasma haemocanis*	5	0.8	0.3 – 1.9
*Rickettsia conorii*	2	0.3	0.1 – 1.2
*Rickettsia massiliae*	1	0.2	0.0 – 0.9
*Rickettsia* spp.	29	4.7	3.3 – 6.7
Total	491	79.4	76.1 – 82.4

CI = Confidence Interval, Cand. M. haematoparvum = *Candidatus* M. haematoparvum, *Cand. M. haemominutum* = *Candidatus* M. haemominutum

**Table 2:** Positivity rates of single and mixed vector-borne pathogens detected in ticks infesting dogs from Chad

	Overall positive		*Rhipicephalus linnaei *(*n* = 430)		*Rhipicephalus muhsamae *(*n* = 177)		*Amblyomma variegatum *(*n* = 6)		*Haemaphysalis leachi (n* = 4)	
Pathogens	*n*	%	*n*	%	*n*	%	*n*	%	*n*	%
Single pathogens (*n* = 267)										
*Anaplasma* spp.	5	0.8	5	1.2	–	–	–	–	–	–
*Anaplasma platys*	7	1.1	7	1.6	–	–	–	–	–	–
*Anaplasma phagocytophilum*	4	0.6	4	0.9	–	–	–	–	–	–
*Ehrlichia canis*	14	2.3	13	3.0	1	0.6	–	–	–	–
*Hepatozoon canis*	205	33.2	161	37.4	43	24.3	1	16.7	–	–
*Rickettsia* spp.	9	1.5	5	1.2	4	2.6	–	–	–	–
*Coxiella*–like endosymbionts	20	3.2	–	–	20	11.3	–	–	–	–
*Rickettsia conorii*	1	0.2	–	–	1	0.6	–	–	–	–
*Rickettsia massiliae*	1	0.2	–	–	1	0.6	–	–	–	–
*Hepatozoon felis*	1	0.2	–	–	1	0.6	–	–	–	–
Double infections (*n* = 84)										
*Anaplasma* spp. + *H. canis*	10	1.6	8	1.9	2	1.1	–	–	–	–
*Anaplasma* spp. + *Coxiella*–like endosymbionts	1	0.2	–	–	1	0.6	–	–	–	–
*A. platys* + *H. canis*	9	1.5	9	2.1	–	–	–	–	–	–
*A. platys* + *A. phagocytophilum*	1	0.2	–	–	–	–	–	–	–	–
*A. platys* + *Cand.* M. haematoparvum	1	0.2	1	0.2	–	–	–	–	–	–
*E. canis* + *H. canis*	29	4.7	28	6.5	1	0.6	–	–	–	–
*H. canis* + *A. phagocytophilum*	1	0.2	1	0.2	–	–	–	–	–	–
*H. canis* + *Rickettsia* spp.	14	2.3	10	2.3	2	1.1	1	16.7	1	25
*H. canis* + *Coxiella*–like endosymbionts	10	1.6	–	–	10	5.6	–	–	–	–
*H. canis* + *Mycoplasma haemocanis*	1	0.2	1	0.2	–	–	–	–	–	–
*H. canis* + *Cand.* Mycoplasma haemominutum	1	0.2	1	0.2	–	–	–	–	–	–
*Rickettsia* spp. + *Coxiella*–like endosymbiont	1	0.2	–	–	–	–	1	16.7	–	–
*Rickettsia* spp. + *Babesia caballi*	1	0.2	–	–	1	0.6	–	–	–	–
*A. platys* + *E. canis*	4	0.6	4	0.9	–	–	–	–	–	–
Triple infections (*n* = 12)										
*Anaplasma* spp. + *A. phagocytophilum* + *H. canis*	1	0.2	–	–	–	–	–	–	–	–
*Anaplasma* spp. + *E. canis* + *H. canis*	2	0.3	2	0.5	–	–	–	–	–	–
*Anaplasma* spp. + *H. canis* + *A. platys*	1	0.2	1	0.22	–	–	–	–	–	–
*Anaplasma* spp. + *H. canis* + *Rickettsia* spp.	1	0.2	–	–	–	–	1	16.7	–	–
*Anaplasma* spp. + *H. canis* + *Coxiella–*like endosymbiont	1	0.2	–	–	1	0.6	–	–	–	–
*A. platys* + *H. canis* + *Rickettsia* spp.	1	0.2	1	0.2	–	–	–	–	–	–
*A. platys* + *H. canis* + *M. haemocanis*	1	0.2	1	0.2	–	–	–	–	–	–
*H. canis* + *Rickettsia* spp. + *Rickettsia conorii*	1	0.2	–	–	1	0.6	–	–	–	–
*H. canis* + *Rickettsia* spp. + *M. haemocanis*	1	0.2	1	0.2	–	–	–	–	–	–
*E. canis* + *H. canis* + *Cand.* Mycoplasma haematoparvum	1	0.2	1	0.2	–	–	–	–	–	–
*E. canis* + *H. canis* + *Cand.* Mycoplasma haemominutum	1	0.2	–	–	–	–	–	–	–	–
Quadruple infections (*n* = 2)										
*Anaplasma* spp. + *A. platys* + *H. canis* + *M. haemocanis*	1	0.2	1	0.2	–	–	–	–	–	–
*A. phagocytophilum* + *E. canis* + *H. canis* + *M. haemocanis*	1	0.2	1	0.2	–	–	–	–	–	–

*–* = no positive detections

**Table 3:** Univariable associations of vector-borne pathogens with tick species and geographic region

						*X*^*2*^ Tests		Fisher’s exact
Pathogen	Variable	Category	*n*	Positive	Prevalence %	*X*^*2*^ statistic *df*	*P-*value^adj^ (Bonferroni)	OR 95% CI
*Anaplasma* spp.	Tick species	*R. linnaei*	430	18	4.2		0.68	0.53
		*R. muhsamae*	177	4	2.3			0.1 – 1.6
	Geographic region	Central	248	9	3.6	1.22 2	1.00	
		North	167	8	4.8			
		South	192	5	2.6			
*Anaplasma platys*	Tick species	*R. linnaei*	430	27	6.3	10.20 1	0.001*	
		*R. muhsamae*	177	–	–			
	Geographic region	Central	248	14	5.6	1.70 2	1.00	
		North	167	5	3.0			
		South	192	8	4.2			
*Ehrlichia canis*	Tick species	*R. linnaei*	430	50	11.6		0.00*	0.1
		*R. muhsamae*	177	2	1.1			0.0 – 0.3
	Geographic region	Central	248	28	11.3	7.78 2	0.24	
		North	167	6	3.6			
		South	192	18	9.4			
*Hepatozoon canis*	Tick species	*R. linnaei*	430	230	53.5	18.51 1	0.00*	
		*R. muhsamae*	177	60	33.9			
	Geographic region	Central	248	120	48.4	0.33 2	1.00	
		North	167	79	47.3			
		South	192	95	49.5			
*Rickettsia* spp.	Tick species	*R. linnaei*	430	17	4.0	0.01 1	1.00	
		*R. muhsamae*	177	8	4.5			
	Geographic region	Central	248	12	4.8	0.55 2	1.00	
		North	167	6	3.6			
		South	192	7	3.6			

*X*^*2*^* statistic* = Chi-square Statistic, *df* = Degrees of Freedom, *X*^*2adj*^* P-value* = Chi-square Adjusted *P*-value

*OR* = Odds Ratio from Fisher’s Exact Test, 95% CI = 95% Confidence Interval

Fisher’s exact test results are provided for cells with expected counts less than 5, as Chi-square tests may not be valid

Significant associations are denoted in bold and with an asterisk

*R. linnaei* = *Rhipicephalus linnaei*, *R. muhsamae* = *Rhipicephalus muhsamae*

## Results

### Dog infestation patterns

A total of 1254 domestic dogs from 56 villages in Chad were examined for tick infestations. The majority of dogs, 85.4% (1071/1254; 95% CI 83.4 – 87.2%), harbored single tick species infestations, although 14.6% (183/1254; 95% CI 12.8 – 16.7%) were coinfested with multiple species. Among dog coinfestations, the presence of two species was most common at 14.35% (180/1254; 95% CI 12.5 – 16.4%), and only three dogs were coinfested with three tick species simultaneously. The combination of *R. linnaei* and *R. muhsamae* was the predominant coinfestation, occurring in 13.6% (171/1254) of all examined dogs. Other combinations were rare: *R. linnaei* + *A. variegatum* (0.6%, 7/1254), *R. linnaei* + *Ha. leachi* (0.2%, 3/1254), *R. linnaei* + *Hy. truncatum* (0.2%, 3/1254), *R. muhsamae* + *A. variegatum* (0.2%, 2/1254), and *R. muhsamae* + *Hy. truncatum* (0.2%, 2/1254). The southern region hosted all five species detected, while the north and central each contained two to three species at the time of sampling.

### Tick species composition

*Rhipicephalus linnaei* was the predominant species at 89.8% (3805/4238; 95% CI 88.7 – 90.8%) with similar proportions of males 50.2% (1909/3805; 95% CI 48.6 – 51.8%) and females 46.2% (1759/3805; 95% CI 44.6 – 47.8%), along with small proportions of nymphs 3.0% (114/3805; 95% CI 2.4 – 3.6%) and larvae 0.6% (23/3805; 95% CI 0.3 – 0.9%). The second most abundant species was *R. muhsamae* at 9.4% (400/4238; 95% CI 8.4 – 10.4%), with more females 59.8% (239/400; 95% CI 54.8 – 64.7%) compared to males 39.5% (158/400; 95% CI 34.7 – 44.5%). The three remaining species were collected in substantially smaller numbers: *A. variegatum* at 0.6% (26/4238; 95% CI 0.3 – 0.8%), *Ha. leachi* at 0.1% (4/4238; 95% CI 0.0 – 0.3%), and *Hy. truncatum* at 0.1% (3/4238; 95% CI 0.0 – 10.2%).

### Phylogenetic analysis of ticks

**Fig. 2:**
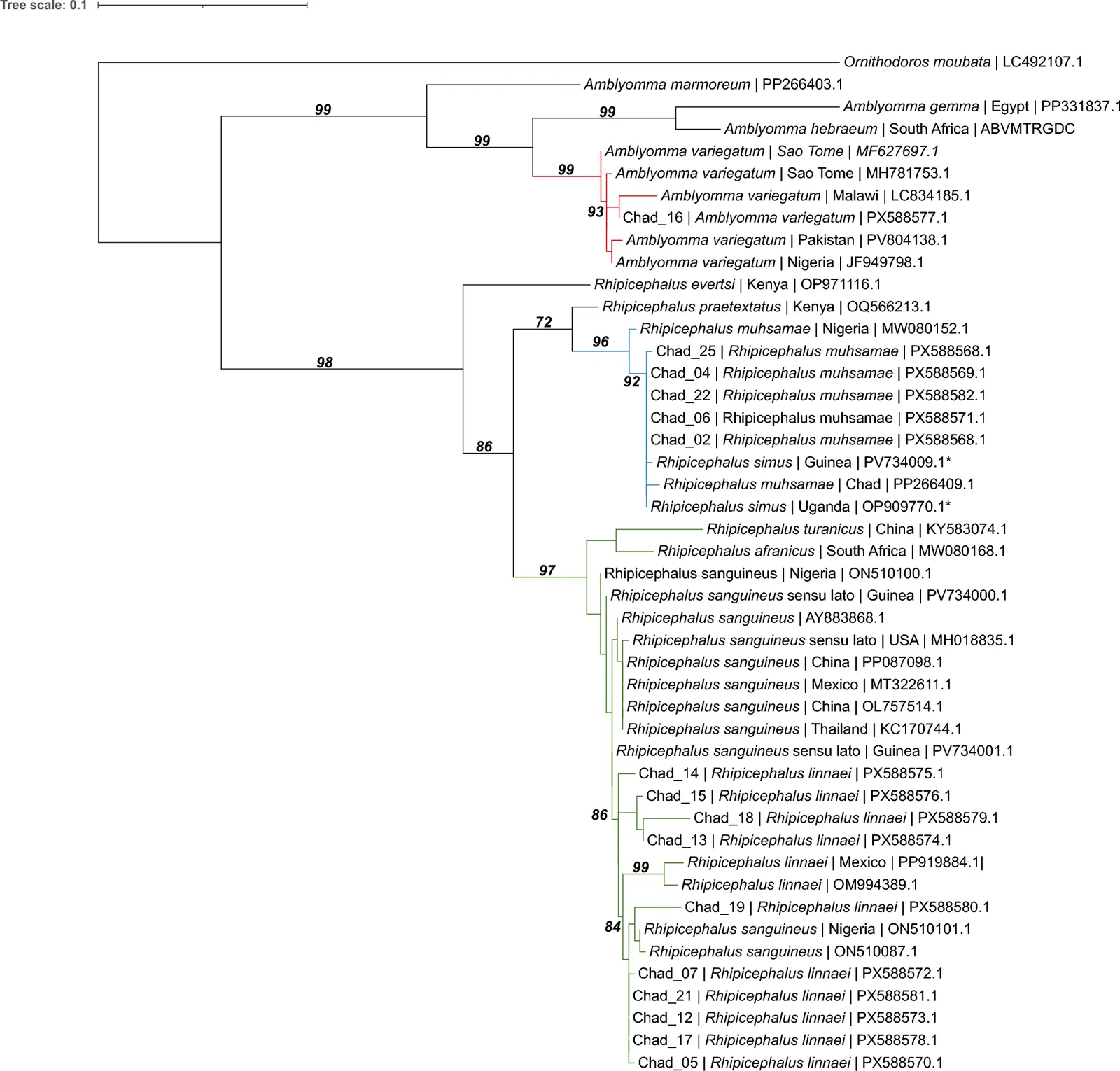
Maximum-likelihood phylogenetic analysis of partial 16S rDNA gene sequences for *Rhipicephalus* spp. and *Amblyomma variegatum* (Chad_16) specimens collected from domestic dogs in Chad. Two *Rhipicephalus simus*; accession numbers PV734009 from Guinea and OP909770 from Uganda (denoted with an asterisk*) appear to be misidentifications as *R. simus* sensu stricto is confined to southern Africa based on Walker *et al*. 2000 [29]

Maximum-likelihood analysis of partial 16S rRNA gene sequences resolved two ixodid genera; *Rhipicephalus* and *Amblyomma* (Fig. 2). The majority of Chadian *Rhipicephalus* samples (shown in green branches, *n* = 10) clustered within a well-supported monophyletic clade (bootstrap 97%) comprising *R. sanguineus* s.l. sequences from diverse geographic origins (Fig. 2). The 16S rRNA evolutionary divergence among these *R. linnaei* specimens in our collection ranged from 0.00 to 0.04 substitutions per site, corresponding to approximately 96 – 100% pairwise sequence similarity, indicating that these ticks represent a genetically homogeneous group consistent with intraspecific variation. A separate, distinct clade comprising five sequences (shown in blue) clustered alongside *R. muhsamae* reference sequences (bootstrap 96%), confirming species-level differentiation. The evolutionary divergence ranged from 0.00 to 0.07 substitutions per site corresponding to approximately 93 – 100% pairwise sequence similarity. A single *A. variegatum* sequence (red) formed a well-supported clade (bootstrap values = 99%) with conspecific African *A. variegatum* sequences. In pairwise comparisons, nucleotide similarity between this sample and homologous sequences from GenBank used in our phylogenetic analysis, ranged from 98.4 to 99.3%. The 16S rDNA sequences obtained were deposited in NCBI GenBank under accession numbers PX588568 – PX588583.

**Fig. 3:**
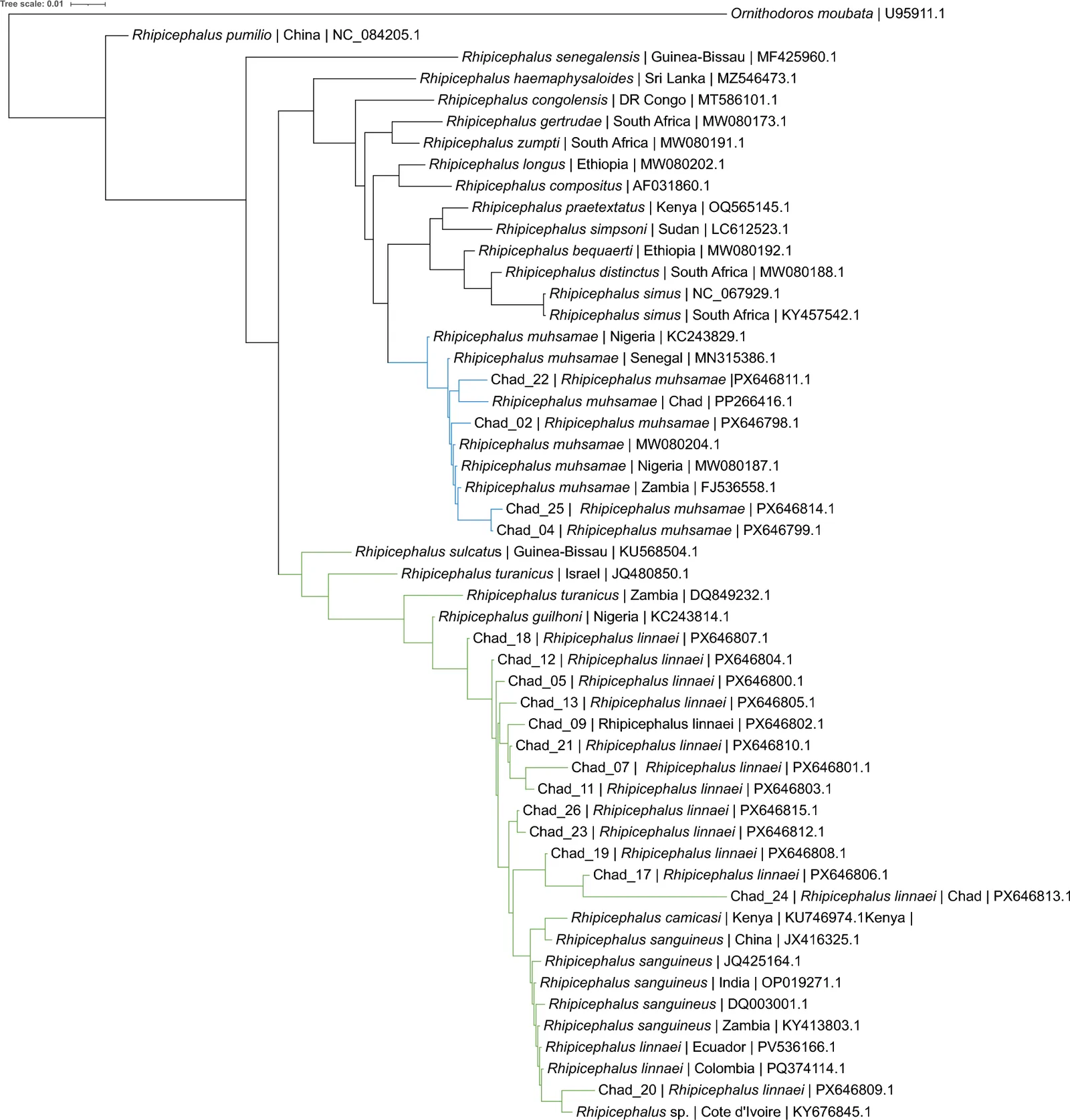
Maximum-likelihood phylogenetic analysis of partial 12S rDNA gene sequences for *Rhipicephalus* spp. collected from domestic dogs in Chad

Phylogenetic analysis based on partial 12S rDNA sequences of *Rhipicephalus* ticks corroborated species identifications obtained from 16S rDNA sequences. The majority of *Rhipicephalus* spp. samples (*n* = 14, shown in green clustered with *Rhipicephalus linnaei* reference sequences, forming a well-supported monophyletic clade (bootstrap 99%) (Fig. 3). This clade exhibited minimal genetic divergence (< 0.08 substitutions per site), consistent with intraspecific variation observed in the *R. sanguineus* species complex [47]. Four additional Chadian samples (shown in blue branches) formed a distinct clade with *R. muhsamae* sequences from West and Central Africa (bootstrap 96%), demonstrating species-level differentiation (Fig. 3). Sequences were deposited in GenBank under accession numbers PX646798 – PX646815.

A single *Hyalomma truncatum* *COI* sequence was obtained, with the top BLAST match (99.69% nucleotide identity) corresponding to *COI* sequences from Cameroon. The sequence was deposited in GenBank under the accession number PX588549. Amplification of the 16S rDNA, 12S rDNA, and *COI* gene regions was attempted for all four *Haemaphysalis leachi* specimens; however, amplification attempts were unsuccessful, likely due to compromised DNA integrity. Species identification for these specimens therefore relied exclusively on morphological characterization. *COI* sequences were not generated for the remaining tick species, as species-level identification was successfully achieved using the 16S rDNA and 12S rDNA markers.

### Pathogen DNA in ticks

#### Single pathogens

Among the 618 ticks tested, 59.1% (365/618; 95% CI 55.1 – 62.9%) were positive for at least one pathogen (Table 2). A total of 491 pathogen occurrences were recorded across these ticks (79.4%; 95% CI 76.1 – 82.4%), exceeding the number of positive ticks due to co-infections in which individual ticks harbored more than one pathogen simultaneously (Table 1). *Hepatozoon canis* was the most prevalent pathogen detected at 46.7% (294/618; 95% CI 43.7 – 51.5%) and present across all tick species and geographic regions (41.1 – 55.9%) (Supplementary table S1). Pathogen positivity rates are shown in Table 1. Notably, *Babesia caballi, Mycoplasma* spp., *Hepatozoon felis, Rickettsia conorii,* and *Rickettsia massiliae*, were each detected at prevalences < 1%. The single *Hy. truncatum* included tested negative for all pathogens.

### Coinfection patterns

Coinfections were frequently observed, with 84 ticks (13.6%, 95% CI 11.1 – 16.5%) harboring dual infections, 12 ticks (1.94%, 95% CI 1.1 – 3.4%) carrying triple infections, and 2 ticks (0.3%, 95% CI 0.1 – 1.2%) with quadruple infections. *Hepatozoon canis* was present in all quadruple and triple coinfections, and the majority (53.3%) of dual coinfections, demonstrating its role as the predominant coinfecting pathogen (Table 2). The most frequent coinfection was *E. canis* + *H. canis* in 4.7% (29/618; 95% CI 3.3 – 6.7%) of ticks, occurring almost exclusively in *R. linnaei* (96.6%, 28/29, 95% CI: 83.8 – 99.4%). Among triple infections, *Anaplasma* spp. + *A. phagocytophilum* + *H. canis* and *Anaplasma* spp. + *E. canis* + *H. canis* were the most common patterns seen in *R. linnaei* (14.3%, 2/14, 95% CI 4.0 – 39.9%). Both quadruple infections involved *H. canis* with multiple *Anaplasma* spp. and were detected in *R. linnaei*. A comprehensive list of all coinfection combinations and their frequencies are detailed in Table 2.

### Factors associated with pathogen detection in ticks

Univariable analysis revealed significant associations between tick species and detection of *A. platys* (*χ*^2^ = 10.20, *df* = 1, *P* = 0.001), *E. canis* (*χ*^2^ = 16.32, *df* = 1, *P* = 0.001), and *H. canis* (*χ*^2^ = 18.51, *df* = 1, *P* = 0.00) (Table 3). After adjusting for covariates, multivariable logistic regression revealed that *R. muhsamae* had significantly lower odds of harboring *E. canis* (OR = 0.1, 95% CI 0.0 – 0.3, *P* = 0.002) and *H. canis* (OR = 0.4, 95% CI 0.3 – 0.6, *P* < 0.001) compared to *R. linnaei*. Ticks from the north had 70% reduced odds of infection with *E. canis* (OR = 0.3, 95% CI 0.1 – 0.6, *P* = 0.014) compared to those from the central part. Geographic region was not significantly associated with *E. canis* in univariable analysis (*P* = 0.24) but was significant in the multivariable model (*P* = 0.014), indicating confounding by tick species. The coefficient estimates, odds ratios, and *P*-values for our logistic regression model are summarized in Table 4.

**Table 4:** Results of multivariable logistic regression model for vector-borne pathogens in ticks infesting dogs from Chad

	Variable	Categories	Estimate	*SE*	OR _*adj*_	95% CI OR	*P-*value _*adj*_
*Anaplasma* spp.	Intercept		− 3.10	0.36	0.05	0.02 – 0.09	< 0.001
	Tick species	*R*. *linnaei*	Reference				
		*R. muhsamae*	− 0.65	− 0.56	0.5	0.1 – 1.4	0.98
	Geographic region	Central	Reference				
		North	0.25	0.50	1.3	0.5 – 3.4	1.00
		South	− 0.39	0.57	0.7	0.2 – 2.0	1.00
*Anaplasma platys*	Intercept		− 2.37	0.28	0.0	0.1 – 0.2	< 0.001
	Tick species	*R. linnaei*	Reference				
		*R. muhsamae*	− 16.91	802.77	0.0	NA	1.00
	Geographic region	Central	Reference				
		North	− 0.78	0.54	0.5	0.1 – 1.2	0.59
		South	− 0.46	0.46	0.6	0.2 – 1.5	1.00
*Ehrlichia canis*	Intercept		− 1.6	0.2	0.2	0.1 – 0.3	< 0.001
	Tick species	*R. linnaei*	Reference				
		*R. muhsamae*	− 2.52	0.73	0.1	0.0 – 0.3	0.002*
	Geographic region	Central	Reference				
		North	− 1.36	0.47	0.3	0.1 – 0.6	0.014*
		South	− 0.34	0.32	0.7	0.4 – 1.3	1.00
*Hepatozoon canis*	Intercept		0.15	0.14	1.1	0.8 – 1.5	1.00
	Tick species	*R. linnaei*	Reference				
		*R. muhsamae*	− 0.80	0.19	0.4	0.3 – 0.6	0.001*
	Geographic region	Central	Reference				
		North	− 0.08	0.20	0.9	0.6 – 1.4	1.00
		South	0.02	0.19	1.0	0.7 – 1.5	1.00
*Rickettsia* spp.	Intercept		− 3.02	0.34	0.0	0.0 – 0.1	1.00
	Tick species	*R. linnaei*	Reference				
		*R. muhsamae*	0.11	0.44	1.1	0.4 – 2.6	1.00
	Geographic region	Central	Reference				
		North	− 0.30	0.51	0.7	0.3 – 1.9	1.00
		South	− 0.29	0.49	0.8	0.3 – 1.9	1.00

*SE* = Standard Error, *OR* = odds ratio, *OR*_*adj*_ = Adjusted Odds Ratio

Bold values with asterisk denote the statistically significant associations

*R*. *linnaei* = *Rhipicephalus linnaei*, *R*. *muhsamae* = *Rhipicephalus muhsamae*

## Discussion

This study contributes data on tick diversity, tick-borne pathogen prevalence, and associated risk factors in domestic dogs from Chad, a region where epidemiological information on ticks and VBPs remains scarce. Although ticks in this study were collected from dogs, the detection of several zoonotic pathogens informs us that humans might be at risk of contracting VBPs in Chad. Employing the tNGS technique allowed the detection of a broad spectrum of pathogens, including the detection of hemotropic mycoplasmas, an occurrence often missed by conventional methods such as PCR and serology.

Based on morphological and phylogenetic analysis, our study documented the predominance of *R. linnaei* (89.8%; 3805/4238) on dogs, followed by *R. muhsamae* (9.4%; 400/4238), with limited detections of *A. variegatum*, *Ha. leachi*, and *Hy. truncatum*. These tick species exhibit broad host ranges encompassing domestic dogs, livestock (particularly cattle), wildlife, and humans as accidental hosts, with significant veterinary and public health implications. The detection of *A. variegatum, Hy. truncatum* and* R. muhsamae* on dogs in this study, despite their primary association with large African mammals (e.g., livestock and wildlife), reflects a potential broader host preference where domestic dogs, livestock, and wildlife coexist, a characteristic of agropastoral ecosystems [48].

The overwhelming predominance of *R. linnaei* aligns with previous reports identifying this species as the most widespread ectoparasite of dogs in tropical and subtropical regions [49], including a recent report from Nigeria, (a neighboring country of Chad) where *R. linnaei* was the sole lineage detected [50].

The presence of *R. linnaei* has been reported to overlap geographically with most of the pathogens detected in this study, including *A. platys, E. canis, H. canis,* and multiple spotted fever group *Rickettsia* species (e.g., *R. conorii, R. massiliae*) [51–53]. The detections of Mycoplasmataceae bacteria (*M. haemocanis*, *Cand*. M. haematoparvum, and *Cand*. M. haemominutum) in *R. linnaei* ticks occurred at a lower prevalence than in the blood of domestic dogs from Chad, as previously documented by our group using tNGS [27]. Taken together, these findings indicate that hemotropic mycoplasmas are circulating in this region but remain largely unrecognized. While *R. linnaei* remains a suspected vector of hemotropic mycoplasmas, mounting evidence demonstrates that canine transmission occurs primarily through non-vector mediated routes, including vertical transmission, direct blood contact, and percutaneous exposure during aggressive interactions such as dog fights [54–56]. As such, observations indicate that infection rates tend to be higher in canine blood than in tick vectors [57]. The dogs from which ticks were collected are primarily outdoors, which likely increases exposure to multiple transmission routes, including contact with infected arthropods, wildlife reservoirs, and other dogs. Hemotropic mycoplasmas are associated with severe anemia in dogs and may exacerbate disease when coinfecting with other VBPs [58].

Together with *R. sanguineus* s.l., the yellow tick, *Ha. leachi,* is also adapted to feeding primarily on domestic dogs, and both are the main species found on dogs in the Afrotropical region [17]. The *Ha. leachi* group comprises multiple cryptic species, such as *Ha. leachi* sensu stricto, *H. elliptica* (South African yellow dog tick), *Ha. moreli*, *Ha paraleachi*, *Ha. punctaleachi*, *Ha. muhsamae, Ha. colesbergensis, and Ha. oliveri* [59, 60]. These species are known to vector *Babesia rossi*, the most virulent canine *Babesia* in Africa, and have been associated with *Rickettsia conorii,* the causative agent of Mediterranean spotted fever in humans throughout Africa [61–63]. Although one *Ha. leachi* was co-infected with *H. canis* and *Rickettsia* spp., no *Babesia* spp. were detected in six ticks screened in this study. Molecular surveys across sub-Saharan Africa reveal variable *B. rossi* prevalence: 23.1% in Malawi [64], 7.7% in Kenya [65], 7.8% in Uganda [66], and over 39% in South African veterinary hospitals [67]. The absence of *B. rossi* in our study is likely attributable to the limited number of dogs that seem infested with *Ha. leachi* or potential geographic variation in infection prevalence, highlighting the need for expanded surveillance to elucidate the epidemiological significance of *Ha. leachi* in the region.

The detection of *Rhipicephalus muhsamae* on dogs is significant as this understudied tick typically infests large ungulates (e.g., cattle, sheep, goats, wildlife) across sub-Saharan Africa, with few reports in domestic dogs [10, 68]. *Rhipicephalus muhsamae* is not currently known to transmit any pathogens, but previous studies have isolated the DNA of various pathogens from this species [9, 21]. The detection of zoonotic rickettsiae (*R. conorii* and *R. massiliae*) in *R. muhsamae* aligns with recent findings by Osip *et al*. [9], which documented six *Rickettsia* spp. (43.3% prevalence) in ixodid ticks from Chadian dogs, with the highest diversity of *Rickettsia* in *R. muhsamae* [9]. Similar findings of *Rickettsia massiliae* in *R. muhsamae* have been reported from Mali [21]. Our tNGS approach detected unidentified *Rickettsia* spp. (5.18%), which could not be resolved to species level, likely due to the short-read lengths or divergent lineages with insufficient representation in reference databases. Although the detection of a microorganism in an arthropod does not necessarily indicate vector competence [69], this finding highlights the need for expanded genomic surveillance using unbiased NGS panels and further investigations of *R. muhsamae*, whose vectorial competence remains unclear [17]. The presence of these bacteria in *R. muhsamae* may also result from co-feeding, a process in which infected and uninfected ticks feed in proximity on the same host, facilitating the immediate exchange of pathogens between vectors without systemic infection of the host [70]. Given the multi-host life cycle of some species included in this study (*R. muhsamae, A. variegatum*), ticks could have acquired pathogens from infected hosts during one blood meal and transmitted them to new hosts/species during subsequent feedings at different developmental stages [7]. We emphasize that this phenomenon may similarly explain pathogen detection in other tick species examined herein, for which vector competency remains experimentally unconfirmed.

Additionally*, Coxiella*-like endosymbionts were detected in *R. muhsamae*, suggesting a specific, potentially evolved symbiotic relationship between this tick species and *Coxiella*-related bacteria [71]. Such tick-specific endosymbionts are increasingly recognized as essential components of tick microbiomes with potential effects on tick biology and pathogen transmission capacity that require downstream investigations in this tick species [17, 71].

*Amblyomma variegatum* is a recognized vector for several important pathogens, including *Ehrlichia ruminantium* and *Rickettsia africae* [72]. Although we did not detect *R. africae*, a highly pathogenic species in Africa, most reports of *R. africae* are from *A. variegatum,* with prevalences ranging from 10 to 100% depending on geographic region and tick life stage [9, 73, 74]. A recent study detected *R. africae* in 81% of *A. variegatum* ticks from Chad using nested PCR, underscoring a substantial risk of rickettsiosis in the region [9]. While enabling broader pathogen surveillance, tNGS is known to have reduced sensitivity compared to qPCR for low-abundance targets, possibly due to competition from host and other microbial sequences [26]. Another possible explanation for the lack of detection in our collection is the limited number of *A. variegatum* specimens (0.6%, 26/4,238 overall, and 6 ticks tested), which may have been insufficient for detection, particularly if local infection rates are low. *Hepatozoon canis* was the most prevalent pathogen detected (49.2%), followed by *E. canis* (8.3%), with the highest infection rates observed in *R. linnaei,* followed by *R. muhsamae.* This high detection of *H. canis* in *R. linnaei* is consistent with high prevalence reported across sub-Saharan Africa using PCR methods, where rates up to 98% have been documented in both ticks and dogs [8, 19, 27, 75, 76]. For example, Heylen *et al.* reported *H. canis* prevalence in tick pools ranging from 20.5% (Namibia, urban) to 91.2% (Nigeria, rural), with an overall prevalence of 60.5% across six countries, Ghana, Kenya, Namibia, Nigeria, Uganda, and Tanzania, using a hydrolysis probe-based multiplex qPCR [8]. The significantly higher prevalence of *H. canis* in *R. linnaei* compared to other tick species is consistent with the established role of *R. sanguineus* s.l. as the primary vectors for this pathogen [8]. Detection of *H. canis* in *R. muhsamae* likely reflects acquisition from feeding on infected canine hosts; however, the vector competence of this species has not been evaluated and warrants further investigation.

The predominant detection of *Anaplasma* spp. and *E. canis* in *R. linnaei* aligns with previous reports of *R. sanguineus* s.l. as the proven vector for *A. platys and E. canis* [14, 53, 77]. Recent surveillance studies in Chad have reported *E. canis* infection prevalence between 7.0 and 27.8% in dogs, highlighting the endemicity of this pathogen in the region [19, 27]. Recent studies indicate that *R. sanguineus* s.l. is not a competent vector for *A. phagocytophilum* [14]. Nevertheless, detection of *A. phagocytophilum* (or a closely related species) in *R. linnaei* in our collection (2.10%) aligns with previous studies detecting this pathogen in *R. sanguineus* collected from free-roaming dogs in Egypt and *Hyalomma marginatum* from horses in Tunisia, with prevalence of 13.7% and 2.3%, respectively [78, 79]. However, the absence of these pathogens in other tick genera in the present study contrasts with some reports of Anaplasmataceae detection in diverse tick species, suggesting potential regional differences in vector competence or pathogen-vector associations [80].

Coinfections represent a significant finding in the present study, with 15.9% (*n* = 98/618) of ticks harboring multiple pathogens consistent with rates reported in neighboring Nigeria (11.8%) [75]. The most common coinfection pattern was *E. canis* + *H. canis* 5.0% (*n* = 31/618). These pathogens are reported to share similar transmission dynamics within *R. sanguineus* s.l. populations, as both utilize the same tick vector and exhibit overlapping ecological requirements [81, 82]. Based on the GLMs, tick species was a significant predictor of pathogen infection. *Rhipicephalus muhsamae* exhibited substantially lower infection rates than *R. linnaei*, with reduced odds of carrying both *E. canis* (OR = 0.1, 95% CI 0.0 – 0.3, *P* = 0.002) and *H. canis* (OR = 0.4, 95% CI 0.3 – 0.6, *P* < 0.001). These patterns may reflect differences in vector competence [17], but could also arise from species-specific host use or behavioral ecology, which were not directly evaluated in this study*.* This finding corroborates the established role of *R. linnaei* as the primary vector for both pathogens [13, 83]. Several factors may contribute to the lack of detection of certain pathogens included in the tNGS panel in this study. Pathogens such as *Bartonella, Borrelia, Cytauxzoon, Francisella, and Leishmania* were not detected at either genus or species-specific levels in any ticks analyzed. This can be partially explained by regional epidemiology, as key pathogens like *Borrelia burgdorferi* and *Francisella tularensis* are non-endemic in Chad and are restricted to the Americas [84, 85]. Additionally, some pathogens typically concentrate in tissues rather than blood (e.g., *Bartonella*), whose primary colonization of endothelial tissues with only intermittent bacteremia, limits its detectability through tick blood meals [86]. Low concentrations of genomic DNA of a pathogen, i.e., below the threshold of tNGS detection, may be outcompeted by high levels of host DNA in engorged ticks [86]. Furthermore, pathogens are only molecularly detectable if they are present, survives within the tick through bloodmeal digestion, and reaches concentrations above the assay's detection threshold [7].

We acknowledge several limitations that may influence the interpretation of our findings. First, screening a single tick per dog likely underestimates tick and pathogen diversity and may have missed coinfections present on the same dog host. Second, the convenience sampling design employed was dictated by the concurrent study logistics, limiting habitat and seasonal representation optimal for tick surveillance. For example, sampling was limited to the late rainy season, restricting the ability to assess seasonal changes in tick communities and pathogen transmission, as tick abundance and activity fluctuate substantially throughout the year in relation to temperature, humidity, and rainfall patterns. This also limited the ticks to a small area of Chad along the Chari River so diversity and prevalence may vary across the country. Third, the tNGS assay used in this study is known to have lower analytical sensitivity than pathogen-specific real-time or nested PCRs, which may lead to false negatives and underestimation of true prevalence [26]. Molecular confirmation was unsuccessful for *Haemaphysalis leachi* probably due to compromised DNA integrity due to prolonged alcohol storage. Finally, restricting pathogen screening to female ticks may bias infection estimates, as males and immature stages can also harbor and transmit pathogens, and sex-specific feeding behaviors affect detection probabilities. Future surveillance in Chad should target both on- and off-host ticks, and samples should be collected during both dry and wet seasons. Sampling non-engorged ticks and utilizing both tNGS and targeted qPCR for key pathogens will reduce host DNA interference and improve detection sensitivity.

## Conclusions

While tick specimens in this study were collected from dogs, some recovered species are recognized as generalist feeders that commonly parasitize livestock, wildlife, and occasionally humans. Additionally, some of the identified pathogens pose a significant zoonotic risk. To mitigate the public health and veterinary risks identified here, interventions must be adapted to the realities of rural Chad, where dogs are largely free-roaming working animals and access to veterinary care is limited. First, owner education on ticks and context-appropriate use of affordable ectoparasiticides should be prioritized, drawing on experience from pastoral livestock systems where simple hand-spray acaricide use is common but often sub-optimal [87]. Second, management practices that reduce unsupervised roaming and close contact between dogs, livestock and wildlife around households and watering points could help limit cross-species pathogen transmission while maintaining dogs’ protective roles [48].

## Supplementary Information

Supplementary Material 1.

## Data Availability

Most data generated and analyzed during this study is included in this published article and its supplementary files. The nucleotide sequences generated during the current study are available in the NCBI repository.

## References

[1] Djiman TA, Biguezoton AS, Saegerman C. Tick-borne diseases in Sub-Saharan Africa: a systematic review of pathogens, research focus, and implications for public health. Pathogens. 2024;13(8):697. 10.3390/pathogens13080697. PMC11356977 39204297

[2] Yessinou RE, Koumassou A, Galadima HB, Nanoukon-Ahigan H, Farougou S, Pfeffer M. Tick diversity and distribution of pathogen in ticks collected from wild animals and vegetation in Africa. Pathogens. 2025;14(2):116. 10.3390/pathogens14020116. PMC11858156 40005493

[3] De la Fuente J, Estrada-Pena A, Venzal JM, Kocan KM, Sonenshine DE. Overview: ticks as vectors of pathogens that cause disease in humans and animals. Front Biosci. 2008;13:6938–46. 10.2741/3200. 18508706

[4] Smith BR, Whitehouse‐Tedd K, Yarnell RW, Marginean M, Popa R, Morley A, et al. Direct interactions between livestock guarding dogs and wildlife in a transhumance grazing system. Conserv Sci Pract. 2025;7:e70120.

[5] Cleaveland S, Meslin FX, Breiman R. Dogs can play useful role as sentinel hosts for disease. Nature. 2006;440:605. 10.1038/440605b. 16572146

[6] Halliday JE, Meredith AL, Knobel DL, Shaw DJ, Bronsvoort BM, Cleaveland S. A framework for evaluating animals as sentinels for infectious disease surveillance. J R Soc Interface. 2007;4:973–84. 10.1098/rsif.2007.0237. PMC2394544 17504735

[7] Sonenshine DE, Roe RM. Biology of ticks volume 2. Oxford: Oxford University Press; 2014.

[8] Heylen D, Day M, Schunack B, Fourie J, Labuschange M, Johnson S, et al. A community approach of pathogens and their arthropod vectors (ticks and fleas) in dogs of African Sub-Sahara. Parasit Vectors. 2021;14:576. 10.1186/s13071-021-05014-8. PMC8594167 34784947

[9] Osip S, Friedman M, Haynes E, Coker SM, Bryan Ii JA, Sidouin M, et al. Prevalence and diversity of spotted fever group *Rickettsia* species in ixodid ticks from domestic dogs in Chad Africa. Ticks Tick Borne Dis. 2024;15:102405. 10.1016/j.ttbdis.2024.102405. 39427603

[10] Cleveland CA, Friedman M, Thompson AT, Haynes E, Coker SM, Bryan JA, et al. Multi-season survey of ixodid tick species collected from domestic dogs in Chad Africa. Vet Parasitol Reg Stud Rep. 2025;57:101165. 10.1016/j.vprsr.2024.101165. 39855853

[11] Walker A, Walker JB, Keirans JE, Horak IG. The genus *Rhipicephalus* (Acari, Ixodidae): a guide to the brown ticks of the world. CUP; 2000.

[12] Dantas-Torres F. The brown dog tick, *Rhipicephalus sanguineus* (Latreille, 1806) (Acari: Ixodidae): from taxonomy to control. Vet Parasitol. 2008;152:173–85. 10.1016/j.vetpar.2007.12.030. 18280045

[13] Baneth G, Samish M, Alekseev E, Aroch I, Shkap V. Transmission of *Hepatozoon canis* to dogs by naturally-fed or percutaneously-injected *Rhipicephalus sanguineus* ticks. J Parasitol. 2001;87:606–11. 10.1645/0022-3395(2001)087[0606:Tohctd]2.0.Co;2. 11426725

[14] Snellgrove AN, Krapiunaya I, Ford SL, Stanley HM, Wickson AG, Hartzer KL, et al. Vector competence of *Rhipicephalus sanguineus* sensu stricto for *Anaplasma platys*. Ticks Tick Borne Dis. 2020;11:101517. 10.1016/j.ttbdis.2020.101517. PMC7571858 32993937

[15] Dantas-Torres F, Otranto D. Further thoughts on the taxonomy and vector role of *Rhipicephalus sanguineus* group ticks. Vet Parasitol. 2015;208:9–13. 10.1016/j.vetpar.2014.12.014. 25579394

[16] Wengi N, Willi B, Boretti FS, Cattori V, Riond B, Meli ML, et al. Real-time PCR-based prevalence study, infection follow-up and molecular characterization of canine hemotropic mycoplasmas. Vet Microbiol. 2008;126:132–41. 10.1016/j.vetmic.2007.06.018 17656047

[17] Walker A, Bouattour A, Camicas JL, Estrada-Peña A, Horak I, Latif A, et al. Ticks of domestic animals in Africa: a guide to identification of species. Biosci. Rep.; 2014.

[18] Cutler SJ, Vayssier-Taussat M, Estrada-Peña A, Potkonjak A, Mihalca AD, Zeller H. Tick-borne diseases and co-infection: current considerations. Ticks Tick-borne Dis. 2021;12:101607. 10.1016/j.ttbdis.2020.101607. 33220628

[19] Haynes E, Garrett KB, Grunert RKA, Bryan JA 2nd, Sidouin M, Oaukou PT, et al. Surveillance of tick-borne pathogens in domestic dogs from Chad, Africa. BMC Vet Res. 2024;20:417. 10.1186/s12917-024-04267-6. PMC11409619 39294647

[20] Fournier PE, Xeridat B, Raoult D. Isolation of a *Rickettsia* related to Astrakhan fever *Rickettsia* from a patient in Chad. Ann N Y Acad Sci. 2003;990:152–7. 10.1111/j.1749-6632.2003.tb07356.x. 12860619

[21] Parola P, Inokuma H, Camicas J-L, Brouqui P, Raoult D. Detection and identification of spotted fever group Rickettsiae and Ehrlichiae in African ticks. Emerg Infect Dis. 2001;7:1014. 10.3201/eid0706.010616. PMC2631901 11747731

[22] Allison RW, Little SE. Diagnosis of rickettsial diseases in dogs and cats. Vet Clin Pathol. 2013;42:127–44. 10.1111/vcp.12040. 23647393

[23] Maggi RG, Birkenheuer AJ, Hegarty BC, Bradley JM, Levy MG, Breitschwerdt EB. Comparison of serological and molecular panels for diagnosis of vector-borne diseases in dogs. Parasit Vectors. 2014;7:127–35. 10.1186/1756-3305-7-127. PMC3972965 24670154

[24] Sanchez-Vicente S, Tokarz R. Tick-borne co-infections: challenges in molecular and serologic diagnoses. Pathogens. 2023;12:1371. 10.3390/pathogens12111371. PMC10674443 38003835

[25] Gu W, Miller S, Chiu CY. Clinical metagenomic next-generation sequencing for pathogen detection. Annu Rev Pathol. 2019;14:319–38. 10.1146/annurev-pathmechdis-012418-012751. PMC6345613 30355154

[26] Kattoor J, Nikolai E, Qurollo B, Wilkes R. Targeted next-generation sequencing for comprehensive testing for selected vector-borne pathogens in canines. Pathogens. 2022;11:964. 10.3390/pathogens11090964. PMC9503002 36145396

[27] Daniel IK, Hakimi H, Ramos RA, Kattoor JJ, Wilkes RP, Nare RNB, et al. High prevalence of vector-borne protozoa and bacteria in dogs from Chad determined using a targeted next-generation sequencing approach. Sci Rep. 2025;15:28215. 10.1038/s41598-025-13431-3. PMC12318021 40753294

[28] Maharana P, Abdel-Lathif AY, Pattnayak KC. Observed climate variability over Chad using multiple observational and reanalysis datasets. Glob Planet Change. 2018;162:252–65. 10.1016/j.gloplacha.2018.01.013.

[29] Walker JB, Keirans JE, Horak IG. The genus *Rhipicephalus* (Acari, Ixodidae): a guide to the brown ticks of the world. Cambridge University Press; 2000.

[30] H, Hoogstraal. African ixodea. US GPO. 1956.

[31] Sonenshine DE. The biology of tick vectors of human disease. In: Goodman JL, Dennis DT, Sonenshine DE, editors. Tick-borne diseases of humans. Washington (DC): ASM Press; 2005. p. 12–36. 10.1128/9781555816490.ch2.

[32] Anderson JF, Magnarelli LA. Biology of ticks. IDCNA. 2008;22:195–215. 10.1016/j.idc.2007.12.006. 18452797

[33] Hughes VL, Randolph SE. Testosterone depresses innate and acquired resistance to ticks in natural rodent hosts: a force for aggregated distributions of parasites. J Parasitol. 2001;87:49–54. 10.1645/0022-3395(2001)087[0049:Tdiaar]2.0.Co;2. 11227902

[34] Koloski CW, Adam H, Siddiqa A, Bourgeois B, Chilton NB, Gulia-Nuss M, et al. Sexual dimorphism of the Lyme disease bacterium in its tick vector – abundance of *Borrelia burgdorferi* is higher in female than male *Ixodes scapularis* larvae. Ticks Tick Borne Dis. 2025;16:102539. 10.1016/j.ttbdis.2025.102539. 40834820

[35] Lv J, Wu S, Zhang Y, Chen Y, Feng C, Yuan X, et al. Assessment of four DNA fragments (COI, 16S rDNA, ITS2, 12S rDNA) for species identification of the Ixodida (Acari: Ixodida). Parasit Vectors. 2014;7:93. 10.1186/1756-3305-7-93. PMC3945964 24589289

[36] Lorenza B, James EK. Analysis of the systematic relationships among ticks of the genera *Rhipicephalus* and *Boophilus* (Acari: Ixodidae) based on mitochondrial 12S ribosomal DNA gene sequences and morphological characters. J Parasitol Res. 2001;87:32–48. 10.1645/0022-3395(2001)087[0032:AOTSRA]2.0.CO;2 11227901

[37] Folmer O. DNA primers for amplification of mitochondrial cytochrome c oxidase subunit I from diverse metazoan invertebrates. Mol Mar Biol Biotechnol. 1994;3:294–9. 7881515

[38] Kumar S, Stecher G, Li M, Knyaz C, Tamura K. MEGA X: molecular evolutionary genetics analysis across computing platforms. Mol Biol Evol. 2018;35:1547–9. 10.1093/molbev/msy096 PMC5967553 29722887

[39] Katoh K, Standley DM. MAFFT multiple sequence alignment software version 7: improvements in performance and usability. Mol Biol Evol. 2013;30:772–80. 10.1093/molbev/mst010. PMC3603318 23329690

[40] Trifinopoulos J, Nguyen LT, von Haeseler A, Minh BQ. W-IQ-TREE: a fast online phylogenetic tool for maximum likelihood analysis. Nucleic Acids Res. 2016;44:W232–5. 10.1093/nar/gkw256. PMC4987875 27084950

[41] Letunic I, Bork P. Interactive Tree of Life (iTOL) v6: recent updates to the phylogenetic tree display and annotation tool. Nucleic Acids Res. 2024;52(W1):W78–W82. 10.1093/nar/gkae268. PMC11223838 38613393

[42] Camilli Gregory HKD. Applicability of chi-square to 2×2 contingency tables with small expected cell frequencies. Psychol Bull. 1978;85:163–7. 10.1037/0033-2909.85.1.163.

[43] Peduzzi P, Concato J, Kemper E, Holford TR, Feinstein AR. A simulation study of the number of events per variable in logistic regression analysis. J Clin Epidemiol. 1996;49:1373–9. 10.1016/s0895-4356(96)00236-3 8970487

[44] Sperandei S. Understanding logistic regression analysis. Biochem Med. 2014;24:12–8. 10.11613/bm.2014.003. PMC3936971 24627710

[45] Sun GW, Shook TL, Kay GL. Inappropriate use of bivariable analysis to screen risk factors for use in multivariable analysis. J Clin Epidemiol. 1996;49:907–16. 10.1016/0895-4356(96)00025-x. 8699212

[46] Heinze G, Wallisch C, Dunkler D. Variable selection – a review and recommendations for the practicing statistician. Biom J. 2018;60:431–49. 10.1002/bimj.201700067. PMC5969114 29292533

[47] Dantas-Torres F, Latrofa MS, Annoscia G, Giannelli A, Parisi A, Otranto D. Morphological and genetic diversity of *Rhipicephalus sanguineus* sensu lato from the New and Old Worlds. Parasit Vectors. 2013;6:213. 10.1186/1756-3305-6-213. PMC3735430 23880226

[48] Virapin M, Sturaro E, Brunschwig G. Multiple risk analysis at the interface between pastoral livestock systems and wildlife. Pastoralism. 2026;15:1413. 10.3389/past.2025.15413.

[49] Šlapeta J, Chandra S, Halliday B. The “tropical lineage” of the brown dog tick *Rhipicephalus sanguineus* sensu lato identified as *Rhipicephalus linnaei*. Int J Parasitol. 2021;51:431–6. 10.1016/j.ijpara.2021.02.001. 33713653

[50] Elelu N, Bankole AA, Daphne HP, Rabiu M, Ola-Fadunsin SD, Ambali HM, et al. Molecular characterisation of *Rhipicephalus sanguineus* sensu lato ticks from domestic dogs in Nigeria. Vet Med Sci. 2022;8:454–9. 10.1002/vms3.655. PMC8959257 35166463

[51] Dantas-Torres F. Biology and ecology of the brown dog tick *Rhipicephalus sanguineus*. Parasit Vectors. 2010;3:1–11. 10.1186/1756-3305-3-26. PMC2857863 20377860

[52] Foley J, Backus L, López-Pérez AM. Focus on brown dog tick-transmitted Rocky Mountain spotted fever in dogs and people: shared threats and solutions. J Am Vet Med Assoc. 2025;263:293–300. 10.2460/javma.24.11.0756. 39879657

[53] Dantas-Torres F, de Sousa-Paula LC, Otranto D. The *Rhipicephalus sanguineus* group: updated list of species, geographical distribution, and vector competence. Parasit Vectors. 2024;17:540. 10.1186/s13071-024-06572-3. PMC11681662 39731169

[54] Huggins LG, Baydoun Z, Mab R, Khouri Y, Schunack B, Traub RJ, et al. Transmission of haemotropic *Mycoplasma* in the absence of arthropod vectors within a closed population of dogs on ectoparasiticides. Sci Rep. 2023;13:10143. 10.1038/s41598-023-37079-z. PMC10287653 37349533

[55] Lashnits E, Grant S, Thomas B, Qurollo B, Breitschwerdt EB. Evidence for vertical transmission of *Mycoplasma haemocanis*, but not *Ehrlichia ewingii*, in a dog. J Vet Intern Med. 2019;33:1747–52. 10.1111/jvim.15517. PMC6639480 31127669

[56] Compton SM, Maggi RG, Breitschwerdt EB. *Candidatus* Mycoplasma haematoparvum and *Mycoplasma haemocanis* infections in dogs from the United States. Comp Immunol Microbiol Infect Dis. 2012. 10.1016/j.cimid.2012.06.004. 22819621

[57] Beus K, Goudarztalejerdi A, Sazmand A. Molecular detection and identification of hemotropic *Mycoplasma* species in dogs and their ectoparasites in Iran. Sci Rep. 2024;14:580. 10.1038/s41598-024-51173-w. PMC10770070 38182649

[58] Kaewmongkol G, Lukkana N, Yangtara S, Kaewmongkol S, Thengchaisri N, Sirinarumitr T, et al. Association of *Ehrlichia canis*, hemotropic *Mycoplasma* spp. and *Anaplasma platys* and severe anemia in dogs in Thailand. Vet Microbiol. 2017;201:195–200. 10.1016/j.vetmic.2017.01.022. 28284610

[59] Apanaskevich DA, Horak IG. Two new species of African *Haemaphysalis* ticks (Acari: Ixodidae), carnivore parasites of the *H (Rhipistoma) leachi* group. J Parasitol. 2008;94:594–607. 10.1645/ge-1374.1. 18605788

[60] Apanaskevich DA, Horak IG, Camicas J-L. Redescription of *Haemaphysalis* (*Rhipistoma*) *elliptica* (Koch, 1844), an old taxon of the *Haemaphysalis* (*Rhipistoma*) *leachi* group from East and southern Africa, and of *Haemaphysalis* (*Rhipistoma*) *leachi* (Audouin, 1826)(Ixodida, Ixodidae). Onderstepoort J Vet Res. 2007;74:181–208. 10.4102/ojvr.v74i3.122. 17933361

[61] Leisewitz AL, Mrljak V, Dear JD, Birkenheuer A. The diverse pathogenicity of various *Babesi*a parasite species that infect dogs. Pathogens. 2023;12(12):1437. 10.3390/pathogens12121437. PMC10746086 38133320

[62] Dick GWA, Lewis EA. A rickettsial disease in East Africa transmitted by ticks (*Rhipicephalus simus* and *Haemaphysalis leachi*). Trans R Soc Trop Med Hyg. 1947;41:295–326. 10.1016/S0035-9203(47)90068-0. 18898709

[63] Kamani J. Molecular evidence indicts *Haemaphysalis leachi* (Acari: Ixodidae) as the vector of *Babesia rossi* in dogs in Nigeria, West Africa. Ticks Tick Borne Dis. 2021;12:101717. 10.1016/j.ttbdis.2021.101717. 33774482

[64] Chatanga E, Kainga H, Razemba T, Ssuna R, Swennen L, Hayashida K, et al. Molecular detection and characterization of tick-borne hemoparasites and *Anaplasmataceae* in dogs in major cities of Malawi. Parasitol Res. 2021;120:267–76. 10.1007/s00436-020-06967-y. 33225402

[65] Ngoka IT, Mbogo K, Kyallo M, Oduori DO, Pelle R. Genetic detection and phylogenetic relationship of *Babesia* species infecting domestic dogs from select regions in Kenya. Sci Afr. 2021;14:e01010. 10.1016/j.sciaf.2021.e01010.

[66] Proboste T, Kalema-Zikusoka G, Altet L, Solano-Gallego L, Fernández de Mera IG, Chirife AD, et al. Infection and exposure to vector-borne pathogens in rural dogs and their ticks, Uganda. Parasites Vectors. 2015;8:306. 10.1186/s13071-015-0919-x. PMC4460633 26043771

[67] Matjila PT, Leisewitz AL, Jongejan F, Penzhorn BL. Molecular detection of tick-borne protozoal and ehrlichial infections in domestic dogs in South Africa. Vet Parasitol. 2008;155:152–7. 10.1016/j.vetpar.2008.04.012 18502588

[68] Langguth J, Chitimia-Dobler L, Nava S, Pfeffer M. The presence of *Rhipicephalus muhsamae* north of the Sahara. Ticks Tick Borne Dis. 2017;8:605–9. 10.1016/j.ttbdis.2017.04.004. 28483409

[69] Eisen L. Vector competence studies with hard ticks and *Borrelia burgdorferi* sensu lato spirochetes: a review. Ticks Tick Borne Dis. 2020;11:101359. 10.1016/j.ttbdis.2019.101359. PMC7127979 32067949

[70] Gomez-Chamorro A, Hodžić A, King KC, Cabezas-Cruz A. Ecological and evolutionary perspectives on tick-borne pathogen co-infections. Curr Res Parasitol Vector-Borne Dis. 2021;1:100049. 10.1016/j.crpvbd.2021.100049. PMC8906131 35284886

[71] Buysse M, Duron O. Evidence that microbes identified as tick-borne pathogens are nutritional endosymbionts. Cell. 2021;184:2259–60. 10.1016/j.cell.2021.03.053. 33930290

[72] Parola P, Paddock CD, Socolovschi C, Labruna MB, Mediannikov O, Kernif T, et al. Update on tick-borne rickettsioses around the world: a geographic approach. Clin Microbiol Rev. 2013;26:657–702. 10.1128/cmr.00032-13. PMC3811236 24092850

[73] Pillay A, Manyangadze T, Mukaratirwa S. Prevalence of *Rickettsia africae* in tick vectors collected from mammalian hosts in sub-Saharan Africa: a systematic review and meta-analysis. Ticks Tick Borne Dis. 2022;13:101960. 10.1016/j.ttbdis.2022.101960. 35537238

[74] Raoult D, Fournier Pierre E, Fenollar F, Jensenius M, Prioe T, de Pina JJ, et al. *Rickettsia africae*, a tick-borne pathogen in travelers to sub-Saharan Africa. NEJM. 2001;344:1504–10. 10.1056/NEJM200105173442003. 11357153

[75] Kamani J, Lee CC, Haruna AM, Chung PJ, Weka PR, Chung YT. First detection and molecular characterization of *Ehrlichia canis* from dogs in Nigeria. Res Vet Sci. 2013;94:27–32. 10.1016/j.rvsc.2012.07.031. 22925936

[76] Williams BM, Berentsen A, Shock BC, Teixiera M, Dunbar MR, Becker MS, et al. Prevalence and diversity of *Babesia, Hepatozoon, Ehrlichia,* and *Bartonella* in wild and domestic carnivores from Zambia, Africa. Parasitol Res. 2014;113:911–8. 10.1007/s00436-013-3722-7. 24363181

[77] El Hamiani KS, Daminet S, Duchateau L, Elhachimi L, Kachani M, Sahibi H. Epidemiological and clinicopathological features of *Anaplasma phagocytophilum* infection in dogs: a systematic review. Front Vet Sci. 2021;8:686644. 10.3389/fvets.2021.686644. PMC8260688 34250067

[78] M’Ghirbi Y, Yaïch H, Ghorbel A, Bouattour A. *Anaplasma phagocytophilum* in horses and ticks in Tunisia. Parasit Vectors. 2012;5:180. 10.1186/1756-3305-5-180. PMC3453519 22935132

[79] Ghafar MW, Amer SA. Prevalence and first molecular characterization of *Anaplasma phagocytophilum,* the agent of human granulocytic anaplasmosis, in *Rhipicephalus sanguineus* ticks attached to dogs from Egypt. Adv Res. 2012;3:189–94. 10.1016/j.jare.2011.08.002.

[80] Kolo A. Anaplasma species in Africa—a century of discovery: a review on molecular epidemiology, genetic diversity, and control. Pathogens. 2023;12(5):702. 10.3390/pathogens12050702. PMC10222256 37242372

[81] Aktas M, Özübek S. Transstadial transmission of *Hepatozoon canis* by *Rhipicephalus sanguineus* (Acari: Ixodidae) in field conditions. J Med Entomol. 2017;54:1044–85. 10.1093/jme/tjx050. 28399211

[82] Chamsai T, Saechin A, Mongkolphan C, Sariya L, Tangsudjai S. Tick-borne pathogens *Ehrlichia, Hepatozoon*, and *Babesia* co-infection in owned dogs in Central Thailand. Front Vet Sci. 2024;11:1341254. 10.3389/fvets.2024.1341254. PMC11019389 38628944

[83] Aziz MU, Hussain S, Song B, Ghauri HN, Zeb J, Sparagano OA. Ehrlichiosis in dogs: a comprehensive review about the pathogen and its vectors with emphasis on South and East Asian countries. Vet Sci. 2023;10(1):21. 10.3390/vetsci10010021. PMC9863373 36669021

[84] Telford SR, Goethert HK. Ecology of *Francisella tularensis*. Annu Rev Entomol. 2020;65:351–72. 10.1146/annurev-ento-011019-025134. PMC8300880 31600457

[85] Dehio C. Molecular and cellular basis of *Bartonella* pathogenesis. Annu Rev Microbiol. 2004;58:365–90. 10.1146/annurev.micro.58.030603.123700. 15487942

[86] Reifenberger GC, Thomas BA, Rhodes DVL. Comparison of DNA extraction and amplification techniques for use with engorged hard-bodied ticks. Microorganisms. 2022;10:1254. 10.3390/microorganisms10061254 PMC9228219 35744772

[87] Tesfaye T, Abate A. Knowledge, attitude and practices study of acaricide usage and tick control in South Omo Zone pastoral areas, South-Western Ethiopia. Heliyon. 2023;9(6):e17212. 10.1016/j.heliyon.2023.e17212. PMC10277579 37342571

